# Dysregulation of extracellular matrix and Lysyl Oxidase in Ehlers-Danlos syndrome type IV skin fibroblasts

**DOI:** 10.1186/s13023-023-03007-7

**Published:** 2024-01-05

**Authors:** Reece Foehr, Keith Anderson, Owen Dombrowski, Anna Foehr, Erik D. Foehr

**Affiliations:** Kin Therapeutics, 300 Professional Center Drive, Suite #311, Novato, CA 94947 USA

**Keywords:** vEDS, *COL3A1*, Collagen III, CTXIII, Lysyl Oxidase, LOXL2, Fibrosis, Aortic Dissection

## Abstract

**Background:**

Ehlers-Danlos syndrome Type IV (aka Vascular Ehlers Danlos, or vEDS) is a dominantly inherited mutation in the Collagen 3A1 gene (*COL3A1*). The disease is characterized by tissue friability and age-related susceptibility to arterial aneurysm, dissection and rupture as well as uterine and bowl tears. These clinical manifestations result in major surgical intervention and decreased life expectancy. Understanding how mutations in *COL3A1* impact the structure and function of the extracellular matrix (ECM) is important to managing the disease and finding treatments.

**Results:**

Skin fibroblasts from vEDS subjects heterozygous for the p.G588S pathogenic variant in the *COL3A1* gene and a normal individual were cultured and studied. Proteomics analysis identified dozens of upregulated proteins related to extracellular matrix dysregulation that is characteristic of fibrosis. Gene expression libraries from cultured primary fibroblasts were screened for messenger RNA (mRNA) markers of ECM degradation. The proteomics and targeted gene expression array results were largely consistent with dysregulation of the extracellular matrix in vEDS. The data show upregulation of multiple Collagen proteins and genes, other ECM components, and enzymes related to ECM processing and turn-over. vEDS fibroblasts expressed significantly more cross linked C-Telopeptide of Collagen III (CTXIII) than normal fibroblasts, indicative of Collagen III degradation and turn-over. Further, the expression and activity of Lysyl Oxidase (LOX), an enzyme that initiates covalent cross-linking of soluble collagen and elastin into protease resistant fibers, is elevated in vEDS fibroblasts compared to normal fibroblasts.

**Conclusion:**

Together, these findings suggest dysregulated ECM deposition and processing, reminiscent of a state of fibrosis. Therapeutics that target the dysregulated ECM proteins or help replace damaged tissue may improve clinical outcomes.

**Supplementary Information:**

The online version contains supplementary material available at 10.1186/s13023-023-03007-7.

## Background

Ehlers–Danlos syndrome type IV, the vascular type (vEDS), results from mutations in the gene for type III procollagen (*COL3A1)*. Affected patients are at risk for arterial, bowel, and uterine rupture [1]. Clinical diagnosis of vEDS can be made early based on physical observations such as easy bruising, thin skin with visible veins, and characteristic facial features. However, more serious clinical manifestations including rupture of arteries, uterus, or intestines may only arise later in life. Genetic testing is the primary and definitive diagnostic tool. Genetic testing may be triggered by clinical exam, clinical manifestation, or family history. The diagnosis may be confirmed by the demonstration that cultured fibroblasts synthesize abnormal Collagen III. There is no approved treatment for vEDS. Blood pressure medications, namely beta blockers and/or angiotensin II receptor blockers, are used to manage the disease. Celiporolol, a β1 antagonist/β2 agonist is used off-label in Europe and is in clinical trials in the USA for vEDS [2].

A study of over 200 confirmed vEDS individuals described the clinical manifestations of the disease [3]. The author found that complications were rare in childhood with only 25% of the index patients having the first complication by age 20, and more than 80% had had at least one complication by the age of 40. The calculated median survival of the entire cohort was 48 years. Most deaths resulted from arterial rupture. Bowel rupture, which often involved the sigmoid colon, accounted for about a quarter of complications but rarely led to death. Complications of pregnancy led to death in 12 of the 81 women who became pregnant. The types of complications were not associated with specific mutations in*COL3A1*. However, other studies have correlated certain mutations with disease severity. For example, mutations that alter glycine amino acid residues in Collagen III correlate with more severe disease [4, 5].

Autosomal dominant mutations in *COL3A1* cause vEDS. Functional Collagen III forms triple helical fiber-like structures supporting the extracellular matrix. Therefore, one mutant protein can assert a dominant negative effect on the normal proteins within the triple helix. The defective collagen protein disrupts the triple helix formation and alters the deposition of properly functioning connective tissue [6]. Indeed, studies correlating genotype and clinical severity of vEDS suggests that individuals with *COL3A1* deletion mutations (aka null mutations) have less severe manifestations of the disease, because the non-mutant allele can compensate, albeit with reduced Collagen III fibers [7]. Some reports suggest that the abnormal Collagen III fibers are retained within the cell, and others suggest that the abnormal or reduced collagen disrupts the overall structure of the extracellular matrix. Regardless, the weakened tissues ultimately leads to aneurysms, dissections and ruptures of vital organs. Therapeutic strategies that target the dysregulated ECM proteins or help replace damaged tissue may improve clinical outcomes.

## Results

Tissues and cell lines derived from individuals with vEDS disease are ideal to study disease-related cellular phenotypes. Early passages of normal human fibroblasts and individuals with vEDS are used for cell based functional assays, mechanism studies, and drug screening. The two related individuals studied are heterozygous for the p.G588S pathogenic variant in the *COL3A1* gene. Both are male, 18 and 48 years old at the time of diagnosis. The 48 year old subject survived an aortic dissection and was subsequently tested for vEDS along with the related individual. The p.G588S variant, located in coding exon 25 of the *COL3A1* gene, results from a G to A substitution at nucleotide position c1762. Approximately two-thirds of *COL3A1* mutations identified to date have involved the substitution of another amino acid for glycine within the triple-helical domain. Two alterations in the same codon, p.G588D and p.G588V have been previously reported in individuals with vascular Ehlers-Danlos syndrome (Pepin M et al., N. Engl. J. Med. 2000). The normal adult fibroblasts used in this study are from a male donor aged 42.

Fibroblasts derived from the dermal biopsy were studied along with normal human primary dermal fibroblasts. The normal and vEDS fibroblast cells grew similarly and have a spindle-shaped bipolar morphology.

Proteomics analysis of cultured human fibroblasts from normal and vEDS subjects indicate ECM and fibrosis related proteins are dysregulated in vEDS fibroblast cells versus normal fibroblasts. Cell pellets from normal (WT) and vEDS fibroblasts were collected, and lysed. The replicate samples were analyzed by nano LC/MS and analyzed by full scan. Data were analyzed using Scaffold DIA 3.2.1. A Total of 4980 proteins were detected. A Students T-test difference was applied to the intensity values and log2 transformed. A list of 1685 proteins from all categories that had statistically significant (*P* < 0.05) changes between normal (WT) and vEDS fibroblasts is included in Supplemental Table (A) This data set underwent a protein family analysis and the 64 ECM family of protein matches with statistically significant (*P* < 0.05) changes are presented in Supplemental Table (B) Table 1 includes the top 10 ECM related proteins (proteinsymbols) that are significantly (*P* < 0.05) upregulated or down regulated in vEDS fibroblasts versus normal fibroblasts (WTF). Several fibral collagen proteins and extracellular matrix proteins are significantly changed. The proteomics data suggest that some collagens are providing a compensatory effect to make up for dysfunctional Collagen III. Six of the top 10 ECM proteins that are upregulated in vEDS versus normal WT fibroblasts are collagens. COL8A1 tops the list and is an important component of the basement membrane. COL12A1 is a fibral associated collagen that forms structures with Collagen 1. COL2A1 is also a fibral collagen. COL5A1 and COL5A2 are both upregulated in the vEDS fibroblasts. Interestingly mutations in COL5A1 and COL5A2 are associated with classical Ehlers Danlos (Type 3), aka hypermobility EDS, which has many similarities to vEDS. This suggests overlapping functions of COL3A1 and COL5A1, COL5A2.

**Table 1:** Proteomics analysis of vEDS and wild type fibroblasts

Proteins Increased in vEDS			Proteins Decreased in vEDS		
Protein Symbol	Log2 Fold Change	*P*-Value	Protein Symbol	Log2 Fold Change	*P*-Value
COL8A1	3.59	0.00725	ADAMTSL4	4.99	0.01604
LOXL2	2.99	0.00771	SPON2	3.98	0.00132
COL12A1	2.91	0.00319	CLU	3.1	0.00012
COL2A1	2.82	0.00554	THSD4	2.62	0.00061
POSTN	2.79	0.00050	VTN	2.61	0.01925
COL5A2	2.72	0.00972	SERPINE2	2.49	0.01213
BGN	2.57	0.02018	TNXB	2.32	0.01365
FLRT2	2.09	0.01790	TGFBR3	2.2	0.00686
COL3A1	2.02	0.00004	DCN	2.13	0.00438
COL5A1	1.99	0.00349	TIMP1	1.92	0.01442

*See Supplemental Data for additional proteomics data analysis

Other proteins/enzymes that are up or down regulated are involved in matrix remodeling. For example, TIMP1, ADAMTSL4, THSD4, and SERPINE2 are down regulated proteins that are involved in the inhibition of matrix proteases.

Also of interest is the upregulation of the profibrotic factor WNT5A and down regulation of TGFBR3 (a TGFβ decoy receptor) in vEDS fibroblasts versus WT fibroblasts. TGF-β and WNT signaling pathways play important roles in regulating the extracellular matrix.

A targeted array of key genes involved in extracellular matrix degradation was used to compare normal and vEDS subject derived fibroblast gene expression. Briefly, fibroblast cells from normal and vEDS subject were grown, harvested for mRNA, and a cDNA library created. The comparative quantification cycle value method was applied to identify changes in mRNA expression. Multiple house keeping genes (5) were used as a reference to normalize samples.

Fold changes in vEDS samples versus normal fibroblasts are reported (Table 2). Several genes for fibral and anchoring collagens were increased in vEDS samples, while other ECM components and regulatory enzyme encoding genes were decreased in vEDS compared to normal fibroblasts. Overlap in the proteomics profile and gene array profile was observed. For instance, several fibral and anchoring Collagens, FBN, FBLN, ADAMTS/L related proteins, and MMP/TIMP were changed in the same way between the proteomics and gene array. The vEDS fibroblasts had increased matrix metalloproteinases (mRNA and protein). For example, *MMP-1* mRNA is significantly upregulated (~ 7-fold) in vEDS fibroblasts. MMP-1 is a metalloproteinase that degrades extracellular matrix proteins including type I, type III and type VII collagen. In the skin, this enzyme is synthesized by fibroblasts and keratinocytes and it plays a key role in the turnover of dermal collagen.

**Table 2:** Human ECM Degradation qPCR Array

QPCR Summary Fold Change vEDS vs. WT	
mRNA Target Symbol	Fold Change
*MMP1*	6.962342489
*COL11A1*	5.104343495
*COL4A1*	3.584455047
*COL6A3*	3.145581688
*BGN*	2.375276316
*COL6A1*	2.340336341
*DMD*	2.169633723
*BMP4*	2.149695757
*ADAMTS4*	2.092166273
*FN1*	1.92169555
*GAPDH*	1.889499158
*COL7A1*	1.721856545
*COL18A1*	1.625125205
*PCOLCE*	1.553823988
*LAMB1*	1.426785625
*LAMA2*	1.423258809
*BMP1*	1.33745086
*LTBP2*	1.257522633
*LAMB2*	1.234407337
*COL1A1*	1.219734013
*CTSS*	1.217699027
*COL5A1*	1.214205762
*ADAM15*	1.178961982
*LAMA5*	1.178854439
*CAPN2*	1.056289346
*COL1A2*	1.055825812
*MMP2*	1.038080068
*CAST*	0.9890324018
*CEACAM8*	0.9820693563
*COL3A1*	0.9718347934
*TIMP2*	0.9652007999
*NONO*	0.904317416
*SERPINH1*	0.853765881
*ADAMTS5*	0.8496761203
*NCSTN*	0.845181235
*SPP1*	0.8239319581
*HTRA1*	0.8237772923
*LDHA*	0.7774874025
*CAPN3*	0.7771390241
*MMP14*	0.7744722796
*ADAM17*	0.7710375361
*CD44*	0.7684384603
*PPIH*	0.7526935221
*A2M*	0.7406034906
*COL5A2*	0.7331314954
*CAPN1*	0.7302497675
*ADAM10*	0.7239983982
*CASP3*	0.7123353739
*LAMC2*	0.7114149282
*NID1*	0.7039386135
*LUM*	0.6820986187
*SPARC*	0.662943773
*DCN*	0.6558146898
*TIMP1*	0.611488994
*HSPG2*	0.6026032143
*LAMC1*	0.5129034627
*PSEN1*	0.5027737176
*COL14A1*	0.4827554669
*ADAM12*	0.454108097
*VCAN*	0.440161167
*LAMA1*	0.4186142104
*LAMA3*	0.2952541361
*PLG*	0.2678408736
*COL4A5*	0.2537784676
*COL4A3*	0.2223562489
*NTN4*	0.2131865271
*PPC*	0.2114188166
*FBN1*	0.1730133034
*MMP12*	0.1668759578
*LAMB3*	0.1646102454
*CAPN10*	0.1454183852
*ADAMTS1*	0.1435083107
*CAPN9*	0.1182502418
*ACAN*	0.1111956171
*MMP3*	0.01051777272

**Fig. 1:**
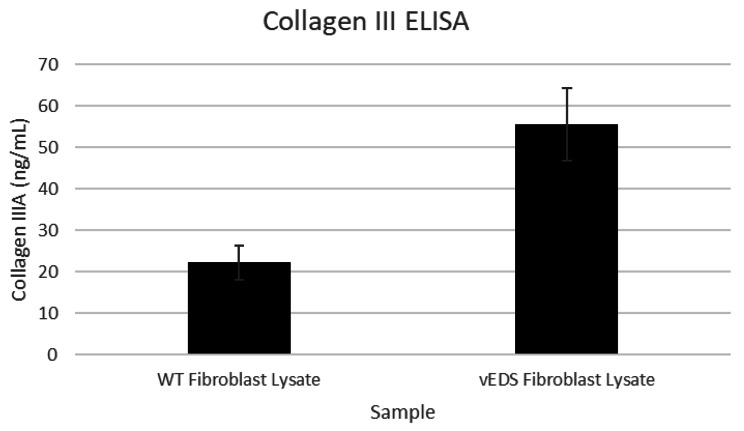
Collagen IIIA protein expression in wild type and vEDS fibroblast cell lysate. Primary human dermal fibrobasts derived from normal (WT) or vEDS individuals were grown in complete media for 2 weeks, washed with PBS and lysed in RIPA buffer and normalized to total protein content. Samples were analyzed for Collagen III protein concentration using commercially available ELISA kit. Average values are reported from triplicate measurements with error bars representing STDEV

**Fig. 2:**
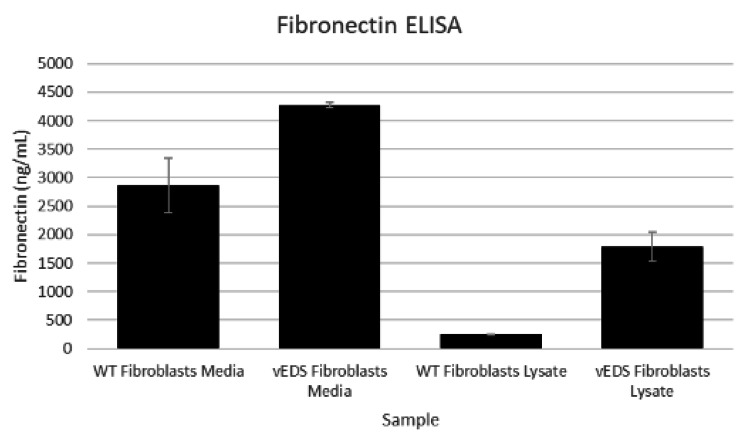
Fibronectin protein expression in wild type and vEDS fibroblast media and cell lysates. Primary human dermal fibrobasts derived from normal (WT) or vEDS individuals were grown in complete media for 2 weeks, the conditioned media collected, and the cells washed with PBS and lysed in RIPA buffer and normalized to total protein content. Samples of conditioned media and lysates were analyzed for Fibronectin protein concentration using commercially available ELISA kit. Average values are reported from triplicate measurements with error bars representing STDEV

**Fig. 3:**
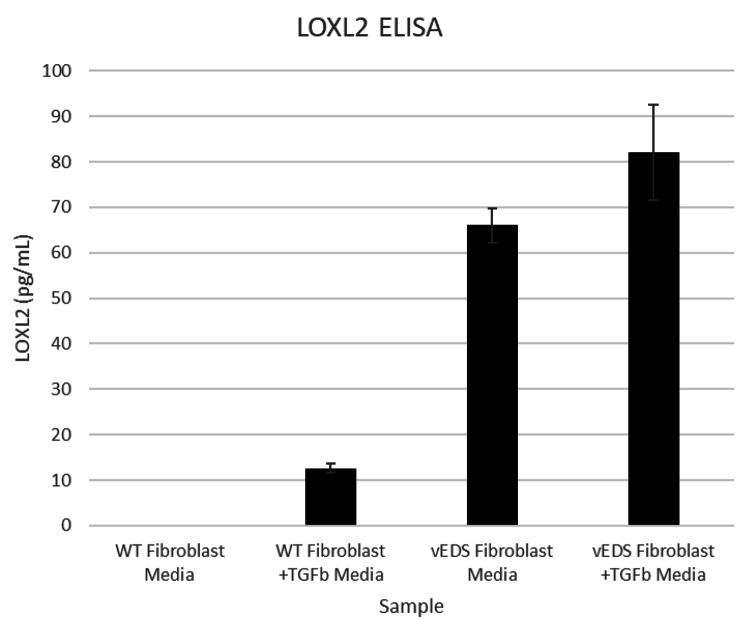
LOXL2 protein expression in wild type and vEDS fibroblast media and cell lysates. Primary human dermal fibrobasts derived from normal (WT) or vEDS individuals were grown in complete media with and without TGFbeta then media was collected. Samples were analyzed for LOXL2 protein concentration using commercially available ELISA kit. Average values are reported from triplicate measurements with error bars representing STDEV

**Fig. 4:**
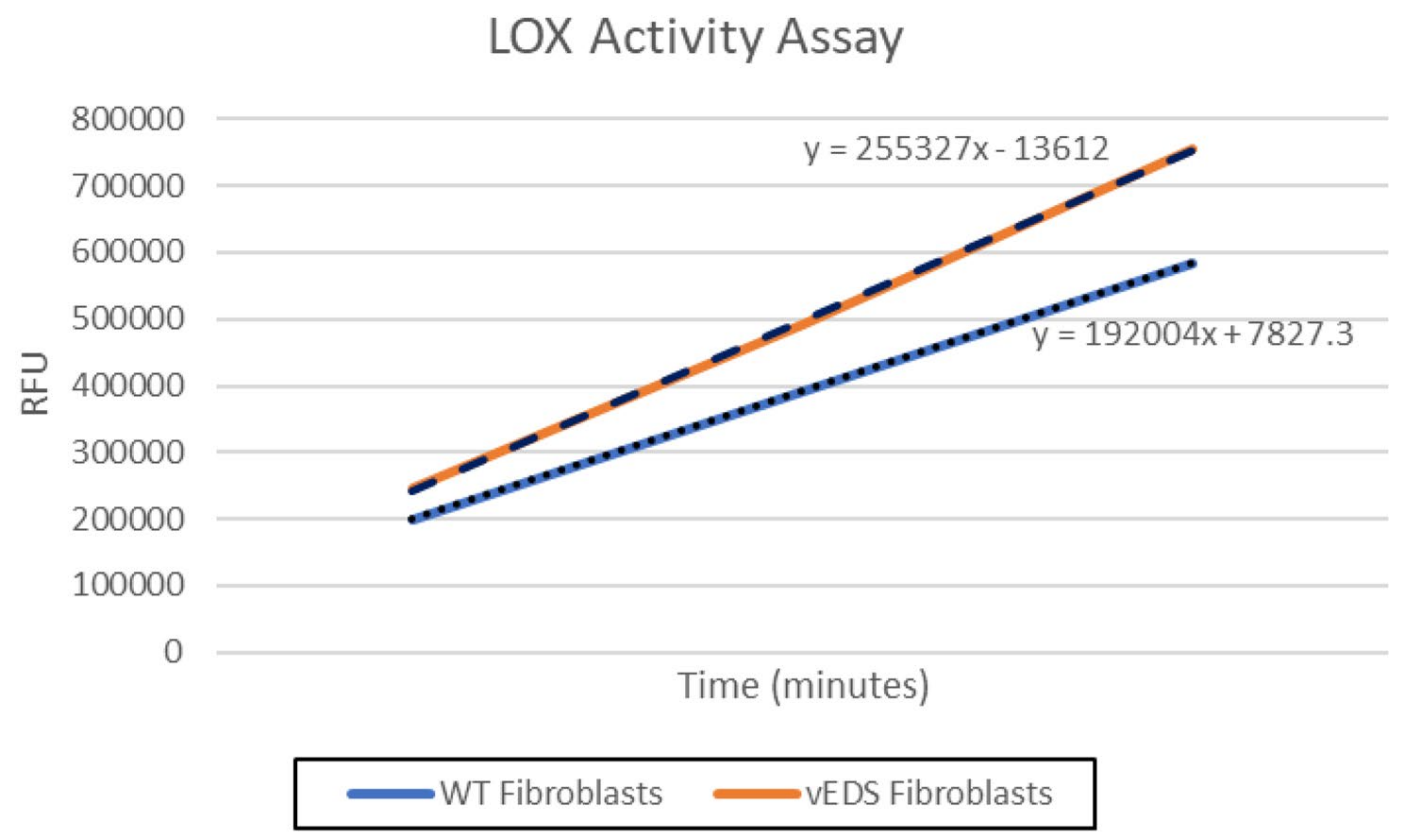
LOX Activity Assay. Conditioned media from primary human fibrobasts derived from normal (WT) or vEDS individuals was collected and tested for LOX activity. The assay uses a LOX substrate that releases hydrogen peroxide upon transformation by the LOX present in the sample. Hydrogen peroxide is then detected over a 30 min period using a red fluorescence substrate for HRP-coupled reactions and measured at Ex/Em = 540/590 nm using a microplate reader (Molecular Devices iD3)

To further investigate the alteration in ECM, we measured Collagen III in both fibroblast cell lysates and conditioned media. These results showed that vEDS fibroblasts had increased levels of Collagen III protein in lysates versus normal fibroblasts but was below quantifiable limit in conditioned cell culture media. This may indicate that mutant and misfolded Collagen III is retained inside the vEDS fibroblasts. (Fig. 1). It is also consistent with the proteomics data that had higher amounts of Collagen III in vEDS fibroblasts versus normal fibroblasts. Whereas the gene expression array did not indicate a significant difference in *COL3A1* gene expression between vEDS and normal fibroblasts (0.97-fold difference). Other genes and proteins related to ECM were altered in vEDS. This includes an increase in Fibronectin (*FN*) as measured in the proteomics array, mRNA expression and by quantitative ELISA (Fig. 2). Similarly, Lysyl Oxidase 2 (LOXL2) protein and LOX activity is increased in vEDS fibroblasts versus normal media (Fig. 3). LOXL2 protein changes were measured by LC/MS and quantitative ELISA. Lysyl Oxidases regulate the ECM in part by cross-linking Collagens and Elastins. We tested lysyl oxidase activity in fibroblast media using a fluorogenic substrate. We showed that vEDS fibroblasts had increased LOX activity. TGFβwas used to upregulate LOXL2 and LOX activity (Figs. 3 and 4, and Table 3).

**Table 3:** LOX Activity Assay

Sample	Vmax (units per second)
Positive Control	15.5
Negative Control	0
WT Fibroblast Media	3.115
WT Fibroblast Media + TGFβ	3.267
WT Fibroblast Media + BAPN	2.841
vEDS Fibroblasts Media	4.431
vEDS Fibroblast Media + TGFβ	4.346
vEDS Fibroblast Media + BAPN	2.258

**Fig. 5:**
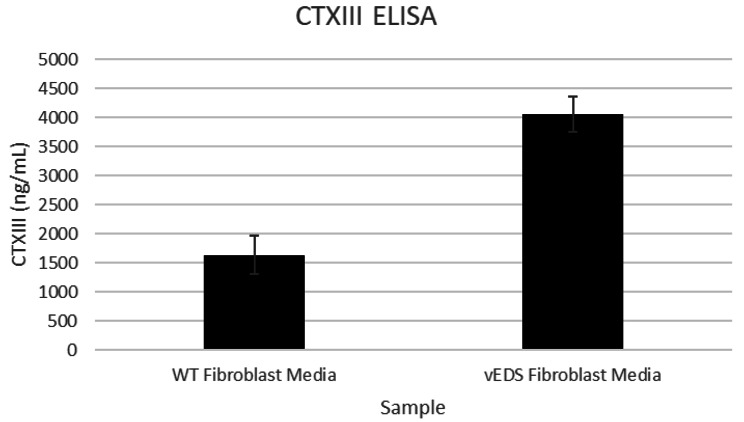
CTXIII Protein in fibroblast cell culture media. Primary human dermal fibroblasts derived from normal (WT) or vEDS individuals were grown and conditioned media collected. Samples were analyzed for CTXIII protein concentration using commercially available ELISA kit. Average values are reported from triplicate measurements with error bars representing STDEV.

The impact of increased LOXL2 protein and LOX activity in vEDS was assessed by measuring cross-linked C-Telopeptide of Type III collagen (CTXIII). Media from normal and vEDS fibroblasts were compared (Fig. 5). These experiments show that CTXIII is significantly increased in vEDS fibroblasts.

LOX activity has been associated with fibrosis and the pathology of certain connective tissues diseases and osteosarcoma. Inhibitors of LOX decrease its activity and thereby reduce the amount of collagen cross-links. We show that BAPN (LOX irreversible inhibitor) reduces LOX activity and reduces the amount of CTXIII in treated fibroblasts (Tables 3 and 4).

**Table 4:** CTXIII Concentration with LOX inhibitor (BAPN).

Sample	CTXIII (ng/mL)
vEDS Fibroblast Media	225
vEDS Fibroblast + BAPN Media	106

## Discussion

Collagen III is critically important for the proper function of extracellular matrix. This is clearly demonstrated by the pathology of vascular Ehlers Danlos Syndrome. Reduced life expectancy due to catastrophic aortic rupture and the multitude of other severe medical conditions observed in vEDS patients underscore the involvement of Collagen III in physiology. The pathogenic mutations in *COL3A1* have a dominant-negative effect (one mutant protein strand may form a triple helix with two normal strands and cause improper folding and function). The mutated protein interacts with normal Collagen III and other ECM components, thereby disrupting tissue formation and cellular function. Much of the mutated Collagen III seems to be retained inside the cells, however, dysfunctional ECM still forms. Skin fibroblasts derived from vEDS patients lack an organized ECM and accumulate cytoplasmic Fibronectin [8]. Indeed, Fibronectin binds fibral collagens and plays an important role in ECM structure and function. Overall, we observed a fibrosis-like profile in the ECM composition of vEDS fibroblasts. This includes upregulation of other collagens, and changes to other ECM components, growth factors, and pro-inflammatory cytokines. Intracellular accumulation of Collagen III has been shown previously [9]. Overly modified Collagen III was measured using polyacrylamide gel electrophoresis [10–12]. Previous studies have explored the abnormal structure of connective tissue and arteries in vEDS patients [13–15]. Some have described aortic tissue from vEDS patients as being moth-eaten, malformed, fragile, rigid, and having characteristics of fibrotic tissue [2, 10, 16]. Therefore, vEDS could be considered as a type of fibrotic disease and thus inform the development of therapeutic interventions.

The hallmark of fibrotic tissue is increased collagen cross-linking. We observed significant increases in cross linked C-telopeptide of Collagen III (CTXIII) in vEDS fibroblasts and concomitant increase in LOXL2 protein and LOX enzyme activity. Collagen cross links increase the rigidity of connective tissues. LOX enzymes play an important role in Collagen cross-linking and should be considered a therapeutic target for vEDS. LOX inhibitors (BAPN) and other more potent and specific LOXL2/LOXL3 inhibitors increase flexibility of soft tissues. However, studies of Marfan Syndrome (a connective tissue disorder due to mutations in *Fibrillin-1*/*FBN*) indicated that neonatal administration of BAPN caused accelerated dilation of the ascending aorta and even premature death in Marfan Syndrome mice. Therefore, understanding the timing, dose, and specificity of LOX inhibitors is important.

Dysregulation of TGFβ and Wnt pathways in vEDS indicate that these pathways could be targets for therapeutic intervention. TGFβ is elevated in plasma obtained from vEDS subjects [17]. This upregulation of TGFβ suggests a role in the pathogenesis of vEDS. TGFβ is known to upregulate LOX enzymes, collagens, and fibronectin. Fibronectin is very important for proper formation of collagen fibrils and ECM deposition. Together, the upregulation of these proteins supports the notion that there is dysregulation of ECM formation in vEDS and compensatory mechanisms at play.

Blood pressure medications are used off-label to treat vEDS. By reducing heart rate and pulsate pressure, celiprolol, a β1 antagonist/β2 agonist, may reduce the mechanical stress on collagen fibers within the arterial wall and be of benefit in patients with vEDS [2]. Beta adrenergic stimulation leads to TGFβ expression and may contribute to the mechanism of action of celiprolol. Other β1 antagonist such as metoprolol are used off label in the US to treat vEDS to reduce pulsate pressure, but do not have β2 agonist activity. In a study using transgenic vEDS mice, celiprolol accelerated rather than reduced death from aortic rupture in both the severe Col3a1G938D/+ and mild Col3a1G209S/+ vEDS mouse models, despite having the predicted effect on pulse rate. Losartan, propranolol, atenolol, amlodipine did not rescue death from aortic rupture in these mice [18].

We also found that WNT5A is increased in vEDS, as evidenced in the proteomics data set. Wnt signaling is an important regulator of LOXL2 expression and collagen cross-linking activity [19]. Wnt5a signaling is profibrotic and therefore, reducing Wnt5a may improve proper ECM formation in vEDS patients.

Another possible treatment for vEDS is the use of stem cells. Stem cells play an important role in vascular formation and regeneration of damaged tissues. Allogeneic stem cells, or stem cells from a matched donor, may repair the damaged tissue and ECM in vEDS patients. Stem cells delivered to the site of damage following dissection, aneurysm, or tissue tare may facilitate repair and replacement of ECM [20, 21]. This strategy has been explored as a treatment for Osteogenesis Imperfecta (OI), or brittle bone disease, caused by dominant negative mutation in Collagen I that causes frequent fractures. The investigators show that mesenchymal stem cells have the ability to migrate, engraft, and differentiate into bone cells in OI patients [22].

## Conclusions

Overall, our findings show dysregulation of ECM deposition and processing in vEDS fibrobasts, is reminiscent of a state of fibrosis. Several compensatory mechanisms seem to be at play in vEDS to deal with the mutation in *COL3A1*. We propose various therapeutic targets amenable to small molecule (e.g. LOX inhibitors) and protein therapeutics (recombinant proteins and antibodies). Other approaches such as cell and gene therapies that target the dysregulated ECM proteins or help replace damaged tissue may improve clinical outcomes for vEDS patients.

## Methods

### Materials

#### Subject material

The two related individuals studied are heterozygous for the p.G588S pathogenic variant in the *COL3A1* gene. Both are male, 18 and 48 years old at the time of diagnosis. The 48 year old subject survived an aortic dissection and was subsequently tested for vEDS along with related individual. The p.G588S variant (also known as c 1762G > A) is located in coding exon 25 of the *COL3A1* gene. The normal adult fibroblasts are from a male donor aged 42.

#### Human collagen III ELISA

The samples were tested using a standard sandwich enzyme-linked immunosorbent assay for the quantitative detection of human Collagen III according to manufactures instructions. (Aviva Systems, Cat#OKEH00269).

#### Human fibronectin ELISA

The samples were tested using an enzyme linked immunosorbent assay for the quantitative detection of human fibronectin in cell culture media according to manufacturers instructions (Invitrogen, Cat #BMS2028).

#### Human cross-linked C-telopeptide of type III collagen (CTXIII) ELISA

The samples were tested according to manufacturers instructions using a sandwich enzyme immunoassay for quantitative measurement of CTXIII in cell culture media using an ELISA kit (MyBioSource, Cat#MBS450320).

#### Lysyl oxidase activity assay

Samples were tested using LOX activity assay kit (Abcam, ab112139) to measure lysyl oxidase activity. The assay uses a LOX substrate that releases hydrogen peroxide upon transformation by the LOX present in the sample. Hydrogen peroxide is then detected using a red fluorescence substrate for HRP-coupled reactions and measured at Ex/Em = 540/590 nm using a microplate reader (Molecular Devices iD3).

#### Human LOXL2 ELISA

Samples were tested using an enzyme linked immunoassay for quantitative measurement of LOXL2 according to manufactures instructions (Biorbyt, Cat# orb563127).

#### LOX inhibitor

3-(methylamino)propanenitrile, lysl oxidase enzyme inhibitor, BAPN (AbCam, Cat#ab144778).

#### Transforming growth factor-beta 1

Human Recombinant (rhTGFB1) was purchased from Lifeome Cat#PYT716.

### Methods

#### Cultured human skin fibroblasts

Skin punch biopsy explants were obtained from two vEDS patients heterozygous for the pathogenic variant p.G588S in the COL3A1 gene (Ambry Genetics). The 4 mm round skin punch biopsy was dissected and transferred onto tissue culture plates containing DMEM with 20% FBS and incubated at 37 C with 5% CO_2_. Primary dermal normal adult human fibroblasts were obtained from ATCC (Cat# PC5-201-012) and cultured in parallel to vEDS patient derived dermal fibroblasts. All tissues used for isolation were obtained under informed consent and conform to HIPAA regulations to protect the privacy of the donor’s Personally Identifiable Information.

#### Proteomics LC/MS/MS

Cell pellets from normal and vEDS fibroblasts were collected, washed with PBS and lysed in 8 M Urea, 50 mM Tris HCl, pH 8 with Roche complete protease. The samples were analyzed by nano LC/MS with Waters M-class HPLC system interfaced to a ThermoFisher Exploris 480 and analyzed by full scan. DIA data were analyzed using Scaffold DIA 3.2.1. A Total of 4980 proteins were detected. Summary statistics are included and analysis of protein groups/family by the Fisher exact test for enriched terms.

#### Human ECM degradation qPCR array

Fibroblast cells from normal and vEDS subject were grown, harvested for mRNA, and a cDNA library created. Samples were added to GeneQuery human ECM Degradation qPCR Array (ScienCell Research Laboratories Cat#GK105) per manufactures directions. The qPCR array, in a 96 well format, with each well containing one primer set that recognizes and efficiently amplifies a specific target genes cDNA. The QPCR products were analyzed using SYBRgreen assay on the QuantStudio 3 system. The quantification method used was Comparative delta-deltaCq (Quantitative Cycle Value Method). Multiple house keeping genes (5) were used as a reference to normalize samples. The data is normalized using the geometric mean of the expression level change, which is equivalent to normalizing using the arithmetic mean of deltaCq of the selected housekeeping genes.

### Electronic supplementary material

Below is the link to the electronic supplementary material.

Supplemental Table A

Supplemental Table B

## Data Availability

The authors declare that the data supporting the findings of this study are available within the paper and its Supplementary Information Tables. Should any raw data files be needed in another format they are available from the corresponding author upon reasonable request.

## Abbreviations

vEDS: Vascular Ehlers Danlos Syndrome
ECM: Exrtracellular Matrix
*COL3A1*: Collagen 3 A1 gene symbol
CTXIII: Cross linked C-Telopeptide of Collagen Type III
